# An Analysis of Clinical and Systemic Factors Associated with Palliative Radiotherapy Delivery and Completion at the End of Life in Alberta, Canada

**DOI:** 10.3390/curroncol30120730

**Published:** 2023-11-21

**Authors:** Siddhartha Goutam, Sunita Ghosh, Jordan Stosky, Alexander Tam, Sarah Quirk, Alysa Fairchild, Jackson Wu, Marc Kerba

**Affiliations:** 1 Faculty of Medicine and Dentistry, University of Alberta, Edmonton, AB T6G 2R7, Canada; 2 Cross Cancer Institute, Edmonton, AB T6G 1Z2, Canada; 3 Department of Oncology, University of Calgary, Calgary, AB T2N 4N2, Canada; 4 Tom Baker Cancer Center, Calgary, AB T2N 4N2, Canada; 5 Department of Physics and Astronomy, University of Calgary, Calgary, AB T2N 1N4, Canada

**Keywords:** palliative radiotherapy, end of life, symptom control, questionnaire, cancer, prognostication

## Abstract

Radiotherapy (RT) is often utilized for symptom control at the end of life. Palliative RT (pRT) may not be taken to completion by patients, thus decreasing clinical benefits and adversely impacting resource allocation. We determined rates of incomplete pRT and examined predictors of non-completion using an electronic questionnaire. **Methods:** A questionnaire was embedded within the RT electronic prescribing system for all five cancer centers of Alberta, Canada, between 2017 and 2020. Prescribing radiation oncologists (ROs) were tasked with completing the questionnaire. Treatment variables were collected for 2040 patients prescribed pRT. Details on pRT courses delivered and completed were used to determine rates of incomplete RT. Electronic medical records of a subset of 367 patients randomly selected from the 2040 patients were then analyzed to examine for association of non-completion of RT with patient, disease, and therapy-related factors. **Results:** Overall, 10% of patients did not complete pRT. The rate of single fractions prescribed as a proportion of all RT fractions increased from 18% (pre-2017: pre-study era) to 29% (2017–2020: study era) (*p* < 0.0001). After conducting multivariate analysis on the overall group, multiple lifetime malignancies (OR:0.64) or increasing the number of pRT fractions (OR:0.08–0.17) were associated with non-completion. Being selected for stereotactic RT (OR:3.75) or survival > 30 days post-RT prescription (OR:2.20–5.02) were associated with greater rates of RT completion. The ROs’ estimates of life expectancy at the time of RT prescription were not predictive of RT completion. In the multivariate analysis of the 367-patient subset, the presence of hepatic metastases (OR 2.59), survival 30–59 days (OR 6.61) and survival 90+ days (OR 8.18) post-RT prescription were associated with pRT completion. Only increasing pRT fractionation (OR:0.05–0.2) was associated with non-completion. **Conclusion:** One in ten patients prescribed pRT did not complete their treatment course. Decreasing pRT fractionation and improving prognostication in patients near the end of life may decrease rates of incomplete RT courses.

## 1. Introduction

Radiotherapy (RT) is an effective treatment modality for symptom palliation in patients with cancer [1]. In the end-of-life (EoL) setting, RT is commonly prescribed to manage symptoms. Palliative RT (pRT) courses may not be taken to completion at the end of life due to the dynamic nature of this clinical setting. Incomplete pRT results in suboptimal symptom control and also has important resource implications to the cancer care system [2,3]. Thus, the proportion of RT that is matched to a patient’s needs and taken to completion is an indicator of quality in cancer care [4].

Courses of single-fraction RT are almost always completed by patients and may also enable improved access to cancer care [5]. Multi-fractionated pRT courses require additional health resources and scheduled visits with increased likelihood of patient non-compliance or patient death prior to therapy completion. Currently, it is thought that a single fraction of RT is appropriate treatment of uncomplicated bone metastasis as well as some spinal cord compressions [6,7]. In other palliative clinical scenarios, treatment and prescribing decisions are dependent on the clinician’s best judgement [7,8]. Investigators have shown differences in ROs’ prescribing practices and that clinicians are inherently imperfect in their prognostication of patient survival [9,10,11,12,13]. Futile therapy is an intervention with a low probability of rendering an expected benefit to patients at a systems level [14,15]. In the context of pRT, we define futile therapy (pRT) as an intervention with a very low probability of providing symptom palliation—specifically RT not completed or RT prescribed in the setting of impending mortality (<30 days before death).

Clinical care and health systems need to be able to determine the factors that predict pRT non-completion. Furthermore, understanding how such factors can be utilized to improve clinical decision making in pRT practice will minimize the mismatch between patients’ RT needs and how RT is prescribed [16,17,18]. To evaluate the impact of current burden of pRT non-completion, its determinants, and prescribing practices, this study utilized a study questionnaire embedded in the radiation oncology information system across five cancer centers in Alberta, Canada. Using questionnaire data, we investigated predictors of RT non-completion and how life expectancy estimates may influence pRT prescribing with the goal of informing future treatment decisions and health care resource utilization. Ethics approval was granted for this project through the Health Research Ethics Board of Alberta (HREBA.CC-16-0973).

## 2. Materials and Methods

### 2.1. Study Design

The Futile Radiotherapy at the End of Life (FutRE) study was launched in November 2017 in Alberta, Canada. It was incorporated into the clinical workflow for each patient within AriaRO^TM^ (Varian, Palo Alto, Santa Clara, CA, USA), the radiation oncology information system, as a routine component of the physician’s prescribing workflow. The questionnaire (Supplemental Figure S1) required the prescribing RO to input the following: the treatment intent (palliative or curative), whether the patient had emergent need for RT (yes/no), the life expectancy of the patient, whether the RT course proposed was retreatment with >80% overlap with a previously treated site (yes/no), whether a single fraction was prescribed (yes/no), and a comment on the rationale of prescribing multi-fraction treatment. The study was initially launched in Alberta Health Services Zones 1, 2 and 3 in November 2017, which were followed by Zone 4 in July 2018 and Zone 5 in September 2018 with data collection for each zone beginning at the time of study implementation.

### 2.2. Study Population

All patients with at least one completed questionnaire between November 2017 and January 2020 were captured from which the palliative population was selected. Patients were excluded from the analysis if residing outside Alberta or younger than 18 years of age. Patients were further categorized as retreated if a treatment site had prior RT (>80% overlap). RT fractions prescribed were grouped into categories of 1, 2–5, 6–10, and 11+ fractions. Stereotactic RT techniques were identified and separated from those receiving non-stereotactic therapy.

### 2.3. Data Collection

For each patient captured, the automated reporting functionality within AriaRO^TM^ was used to aggregate the number of fractions prescribed and completed, the prescribed dosage, treatment site, and whether treatment prescribed was stereotactic body radiotherapy (SBRT/SRT) or stereotactic radiosurgery (SRS). RT prescription data was post-processed as outlined in Appendix A.

Patient demographics and disease data were collected from the Alberta Cancer Registry (ACR) database, and they included age, sex, date of diagnosis, zone of residence, number of lifetime malignancies, vital status, and death date (for deceased patients). Hospitalizations and emergency department visits within 60 days before the initial consultation date and inpatient/outpatient status were captured from the Discharge Abstract Database (DAD) and National Ambulatory Care Reporting System (NACRS).

To establish RT utilization and practice patterns prior to study implementation, registry data and AriaRO^TM^ data were collected retrospectively for patients who had undergone pRT in 2016 and 2017 and died preceding the study implementation in November 2017. This served as a baseline with which to compare the study results.

To examine for associations between pRT completion and clinical factors (unavailable in the AriaRO^TM^ database), clinical data from a convenience sample/random subset of patients (n = 367) from the larger pRT cohort underwent electronic chart review, and this was then linked back with the full dataset. For this subset, Karnofsky performance status, presence of hepatic metastases, brain metastases, administration of palliative chemotherapy, clinical setting (inpatients versus outpatients), presence of anorexia and dyspnea and baseline bloodwork was captured.

### 2.4. Outcomes of Interest

The primary outcome of interest was the proportion of pRT courses taken to completion. RT was considered complete only if the total number of fractions prescribed equaled the number of fractions delivered. Variables of interest were then assessed for their association with this outcome. How pRT fractionation completion rates varied following implementation of the study was examined by assessing the change in proportions over time (pre and post-study implementation).

The second outcome of interest was the association between pRT completion rates and clinical factors.

### 2.5. Study Variables

The variables analyzed for association with the primary outcome were sex, age, number of lifetime malignancies, retreatment, life expectancy estimate, proximity to death, distance to RT site, anatomic site of RT (chest, bone, spine, pelvis, brain), highest RT fractions planned to any site, whether treatment was SBRT/SRS, ED visit/hospital admission 60 days prior to RT consultation, and distance to cancer center. To determine changes in pRT practice prior to and post-study implementation, the proportion of treatments prescribed in each era were categorized by fractionation—1, 2–5, 6–10, and 11+—quantified and compared.

A random subgroup (n = 367) from the overall cohort underwent chart review and was analyzed in a separate multivariable analysis so as to better examine for associations between clinical patient factors and RT completion, which were unavailable for data extraction from the radiotherapy EMR. This included dyspnea, anorexia, PaP score, baseline lab work, presence of hepatic metastases, presence of brain metastases, whether concurrent systemic therapy was being delivered, whether the patient was attending pRT from home, hospital or long-term care, proximity to death, and highest number of pRT fractions prescribed to any given anatomic location.

### 2.6. Statistical Analysis

Descriptive statistics were generated to observe the demographics of the study population. Mean and standard deviations were reported for continuous variables. Frequency and proportions were reported for the categorical variables. Chi square tests were used to measure statistical significance differences in proportions between two categorical variables. The outcome of interest was RT completion (yes/no); hence, binary logistic regression was used to determine the factors associated with the outcome of interest. Univariate association of the study variables discussed above was conducted. Variables significant at the univariate association at the *p* < 0.10 level were considered for the multivariate analysis. Clinically important variables were included in the multivariate analysis irrespective of their statistical significance. Unadjusted univariate odds ratios (ORs) of RT completion were reported for each variable assessed. The most parsimonious model was chosen as the final model. The final multivariate model included variables that were significant at *p* < 0.05 and clinically significant variables. Odds ratios (ORs) and 95% confidence intervals (CIs) along with *p*-values were reported for the univariate and multivariate logistic regression model.

All statistical analyses were conducted using SAS version 9.4 (SAS Institute Inc., Cary, NC, USA) and R version 3.6 (The R Foundation). A *p*-value < 0.05 was used for all statistical significance.

## 3. Results

### 3.1. Demographics

Between 2017 and 2020, 4933 questionnaire responses were collected. Overall, 4330 records pertained to 2166 palliative patients. Of these, 2040 patients were eligible for analysis, while 197 (10%) did not complete radiotherapy.

The questionnaires for these patients were completed by 54 ROs across five radiotherapy centers. The median number of questionnaires completed over the study period by provider was 55.5 (range: 1–450). The proportion of questionnaires completed by each provider that represented single fractions ranged from 0 to 80% across providers with the median proportion being 25.4%. Patient baseline characteristics are described in Table 1.

### 3.2. Logistic Regression Analyses for Factors Associated with pRT Completion

On univariate analysis, six factors were associated with RT completion; multiple lifetime malignancies, RT course being more than 1 fraction, SBRT/SRS delivery, emergency department visit or hospital admission within 60 days before RT consultation, and survival for longer than 30 days after the date of RT prescription. No association was noted between either cancer site or RT site and RT completion rates, which as a result were excluded from the univariate and multivariate models.

In the multivariable analysis, only multiple malignancies (OR: 0.66) receiving 2–5 fractions (OR: 0.17), 6–10 (OR: 0.08), and 11+ fractions (OR: 0.09) were significant (Table 2). Selection for stereotactic RT (OR: 3.75) and proximity to death being 30–59 days (OR: 2.20), 60–89 days (OR:2.61) and 90+ days (OR:5.02) were associated with increased RT completion in both the univariate and the multivariable analysis (Table 2). Age, gender, retreatment, distance to RT site, and prediction of life expectancy were not associated with completion in the adjusted logistic regression model.

The analysis of the clinically enriched patient subset data is presented in Table 3. This shows an association between pRT completion and hepatic metastases and survival for more than 90 days after pRT prescription on univariable analysis. There was a negative association between a maximum prescription of 6–10 fractions to any treatment site and pRT completion in the univariable analysis.

In the multivariable analysis, there was a positive association between pRT completion and hepatic metastases (OR: 2.59) and survival between 30 and 59 days (OR: 6.61) and greater than 90 days after RT prescription (Table 3). The presence of dyspnea, anorexia, RT to any treatment site, brain metastases, and Karnofsky performance score were not associated with RT completion.

### 3.3. Estimates of Life Expectancy

Questionnaire data indicated that 65% of RT prescriptions were given in the context of overestimated LE, 9% were given in the context of underestimated LE, and 26% were given in the context of correctly estimated LE. When LE was overestimated, 30% (95%CI: 28–32%) of prescriptions given were single fractions compared to 39% (95%CI: 33–45%) when LE was underestimated (*p* = 0.002). When LE was correctly estimated, the number of prescriptions of single fractions was 35% (95% CI: 31.4–38.5%).

Overall, 95% of the single fractions prescribed in the last 90 days of life were completed, and only 33% of 11+ fraction therapies in the last 90 days were completed. In the last 30 days of life, 87% of the prescribed single fractions were completed, while only 45% of 6–10 fraction therapies and 0% of 11+ fractions therapies were completed.

### 3.4. Change in pRT Patterns over Time

**Figure 1:**
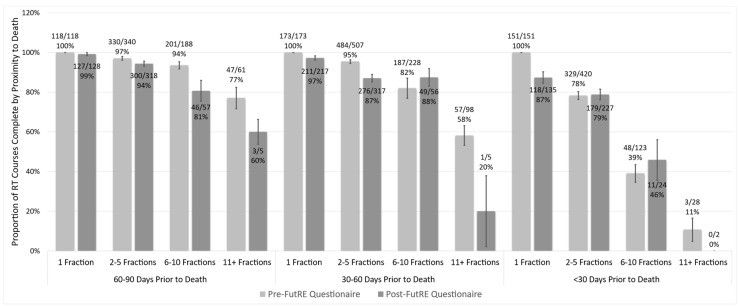
Proportion of prescribed courses taken to completion as a function of fractionation and proximity to death. Proportions gathered prior to and post-study implementation indicate rates of lower completion with increased fractionation with proximity to death. Error bars represent +/− standard error.

Prescriptions in both the pre- and post-study implementation eras showed a decreased completion of pRT with impending mortality when involving multiple fractions (Figure 1). In the pre-study era, 28 of 722 RT prescriptions were for >10 fractions in the last 30 days of life. This proportion was significantly lower in the post-study cohort, where only 2 of 388 prescriptions were for >10 fractions in the last 30 days of life (*p* = 0.001).

**Figure 2:**
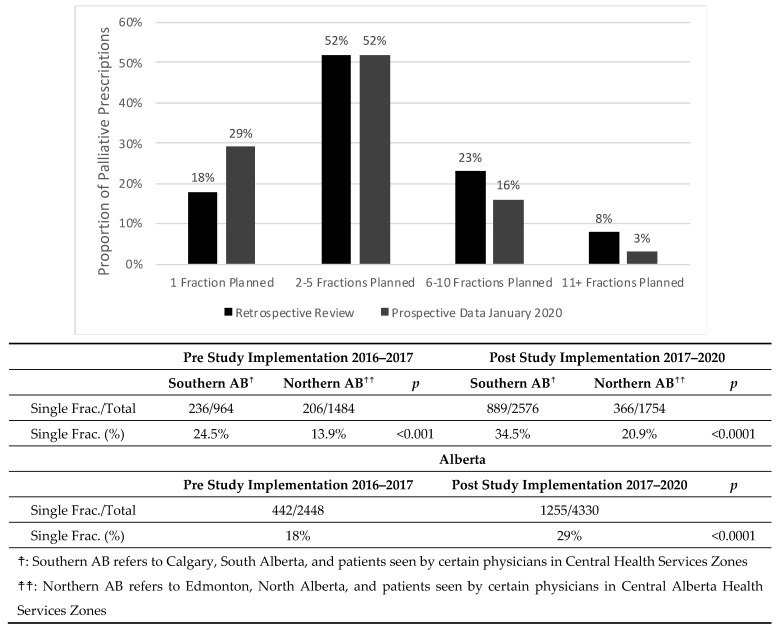
Proportion of cumulative palliative prescriptions in each fractionation category from the pre-study implementation to post-study implementation in January 2020. A comparison of the single fraction proportion across provinces shows statistically significant differences (*p* < 0.00001) between Southern AB and Northern AB both in the pre-study dataset and the post-study dataset. Following the implementation of the study, an increase in proportion of prescribed single fractions has been noted province wide (*p* < 0.00001).

The pre-study prescribing rate was 18% single-fraction, 52% 2–5 fraction, 23% 6–10 and 8% >10 fraction therapies prescribed by ROs in 2016–2017. There was a statistically significant difference in the proportion of single fractions prescribed in both the historical and post-study cohort between Southern Alberta and Northern Alberta cancer centers (Figure 2).

Since the study was implemented, the proportion of single-fraction prescriptions increased to approximately 29% (*p* < 0.0001) of all palliative fractions prescribed, while 2–5 fraction prescriptions have remained stable at 52%. Meanwhile, 6–10 fraction prescriptions decreased to 16% (*p* < 0.0001) and >10 fraction prescriptions have decreased to 3% (*p* < 0.0001).

## 4. Discussion

The primary goal of this study was to evaluate the rates and predictors of pRT non-completion at EoL. Only two factors were associated with pRT non-completion: multiple malignancies and receiving multiple fractions. Being selected for stereotactic RT and survival longer than 30 days was associated with increased RT completion. Single fractions are almost always completed, often the same day. The difference in completion between fractionation categories becomes more pronounced with impending mortality as patients who deteriorate rapidly usually have their pRT stopped.

Overall, 15.7% of patients had multiple lifetime malignancies, and this was associated with RT non-completion. It is possible that this factor is associated with frailty or the psychological well-being/state of the patient, although this study was not designed to assess these factors. A large European study showed a reduction in the five-year relative survival of a cancer cohort when individuals with multiple cancers were included within the cohort [19]. Individuals with multiple cancers may have a greater burden of comorbidities associated with or stemming from their cancers and prior treatments. Combined, these may increase the likelihood of rapid deteriorations in clinical status during their course of RT, resulting in premature discontinuation. Surprisingly, hospitalization at the time of RT consultation was not significantly associated with RT non-completion in the analysis. This was unexpected as inpatients likely represent a more unwell population. Hospital admission within 3 months of palliative RT has previously been described as a predictor of lower life expectancy and an important prognosticator in EoL [20].

Patients prescribed stereotactic body radiotherapy (SBRT) or radiosurgery (SRS) were more likely to complete therapy. SBRT/SRS is more resource intensive; requiring triage, determination of suitability for treatment and a rigorous vetting process involving multidisciplinary rounds. For this reason, patients undergoing SBRT/SRS are under a selection bias and are thus more likely to complete therapy.

Furthermore, we found that pRT completion was not associated with measurable clinical factors that would suggest a deterioration of treatment. The presence of brain metastases and the location of the patient—whether patients accessed pRT from home or hospital—were not associated with discontinuation. The PaP score was also unexpectedly not a factor in RT discontinuation. Only increasing the fractions prescribed was important. It is possible that there are other possible factors that were not captured, including the importance of patient and caregiver preferences at the end of life that may be associated and require further study. Interestingly, PaP has been shown in multiple clinical scenarios to discriminate for survival duration [21,22]. It is possible that the tool itself was designed/validated in patients who were closer to death and is not entirely applicable to decision making around the initiation and continuation of palliative RT. We hypothesize that other factors such as changes in the philosophical approach to care (i.e., optimizing comfort and reducing transportation) may also be factors influencing decision making to stop treatment. This project was not designed to capture the rationale for treatment discontinuation. It is also unclear and beyond the scope of this study to determine whether or not the incomplete courses of pRT delivered were associated with a benefit, were entirely futile, or were potentially even harmful.

When comparing findings from the pre-study period to the study period including the questionnaire rollout and implementation, there was an observed increase in single-fraction RT over time. Studies demonstrate the benefits of short RT courses in various palliative contexts including bone metastasis and spinal cord compressions [6,7,23,24,25]. Surprisingly, we found that in the post-study period, there was a higher rate of single fraction non-completion among patients who died <30 days of prescription. The reason for this finding may perhaps be attributable to issues around patient selection at the time of consult and RT prescription. Although the absolute magnitude is small—involving 17 patients—follow-up on this trend to examine for a systematic change in practice at the end of life would be worthwhile. With our study design, it cannot be ascertained whether the implementation of the questionnaire has impacted clinicians’ decision making around prescribing or whether evolving evidence influencing practice patterns over time are causal for this trend. Our observation of a change in prescribing patterns occurred over a very short period of time (3–5 years) and was inclusive of ROs province-wide with otherwise no new policy changes in the various radiation departments. There is thus also unlikely to be a selection bias in the ROs who prescribed pre and post-study. In other oncology contexts, it has been shown that point-of-care clinical decision support systems can influence and standardize practice [26,27], and in the RT context, similar processes have increased the selection of 1–5 treatment fractions [28].

Surprisingly, estimates of life expectancy by ROs were not shown to be associated with RT completion even when adjusted for RT fractionation. In the decision to offer palliative radiation for patients, ROs will have intrinsically considered a patient’s proximity to EoL in their decision making around the decision to treat and fractions prescribed, creating an inherent selection bias. Events predating CT simulation and pRT prescription were not captured and were unavailable. It is also accepted that physicians have a limited ability to correctly estimate survival. Our study captured some factors pertaining to patient symptoms in the study subset including dyspnea, anorexia, and KPS ≥ 70 or less, and the presence of brain metastases. These factors were not found to be associated with pRT completion. The PaP which captures some lab parameters was also not associated with completion. In practice, it is the prescribing radiation oncologists who are ultimately making decisions about whether pRT may be indicated in patients with lower performance status. There is a “floor” below which bedridden or severely symptomatic patients would be deemed to unwell to tolerate simulation and pRT delivery. Our study was not designed to capture or validate the influence of the prescriber or other factors such as differential cut-offs for performance status (PS) or other laboratory parameters such as albumin. This is currently under investigation by the authors.

One limitation of this study is bias that may have been introduced by the self-selection of those clinicians choosing to complete the questionnaire. The study design did not mandate questionnaire completion and relied on channels of departmental communication to encourage participation by colleagues. Because of the volunteer nature of the questionnaire, a detailed characterization of aspects pertaining to the questionnaire completion was not possible. Reassuringly, at a population level, patients represented by the questionnaire were likely indicative of our usual patient population. This is reflected by the fact that a review of the pre-study era (2016–2017) distribution of the palliative prescription patterns showed a similar distribution of fractionation to the study cohort. This would be unlikely if questionnaire completion was largely concentrated among a small group of subspecialized oncologists with a particular interest in palliative care. Additionally, the overall trend in questionnaire completion when examined for impending mortality was similar in both groups, supporting the likelihood that the questionnaire patient cohort was reflective of the typical palliative RT population seen in our centers.

## 5. Conclusions

Overall, 10% of our palliative patients did not complete their prescribed pRT. Although the clinical impact of such a shortfall may be clinically important, minimizing future mismatches would likely improve resource utilization for patients receiving radiation at the EoL. Our findings identified some factors associated with RT non-completion including fractionation, lifetime malignancies and selection for stereotactic RT. How to best integrate other predictors of survival in patients nearing EoL and for which palliative RT is being considered remains an ongoing challenge to clinical practice.

## Figures and Tables

**Table 1:** **A.** Characteristics of all patients (n = 2040) treated with pRT included in the Futile Radiotherapy at the End of Life (FutRE) study. **B.** Baseline characteristics of a randomized subset of patients (n = 367) treated with pRT who were selected from the overall cohort for electronic chart review.

**(A)**	
**Patient Demographics**	**N(%)**
Gender (n = 2040)	
Male	1085 (53.2%)
Female	955 (46.8%)
Age (n = 2040)	
Age <60	524 (25.7%)
Age 60–79	1123 (55.0%)
Age ≥80	393 (19.3%)
Median	69
Cancer Site (n = 2040)	
Breast	268 (13.1%)
Bronchus/Lung	607 (29.8%)
Prostate	291 (14.3%)
Kidney	74 (3.6%)
Other	800 (39.2%)
RT Treatment Site (n = 2040)	
Head/Brain	248 (12.2%)
Extremity	118 (5.8%)
Chest/Abdo/Pelvis including Spine	1348 (66.1%)
Multiple	326 (16.0%)
Number of Lifetime Malignancies (n = 2040)	
Single	1720 (84.3%)
Multiple	320 (15.7%)
Retreatment (n = 2040)	
No	1800 (88.2%)
Yes	240 (11.8%)
Highest Fractions Prescribed ^☨^ (n = 2040)	
1	388 (19.0%)
2–5	1181 (57.9%)
6–10	391 (19.2%)
≥11	80 (3.9%)
Survival Estimate by Category (n = 2040)	
Less than 1 month	14 (0.7%)
1–3 months	210 (10.3%)
3–6 months	439 (21.5%)
6–12 months	732 (35.9%)
Greater than 12 months	645 (31.6%)
Proximity to Death (n = 2040)	
Less than 30 days	157 (7.7%)
30–59 days	234 (11.5%)
60–89 days	218 (10.7%)
Greater than or equal to 90 days	1431 (70.1%)
Hospitalization or Emergency Department Visit 60 Days Prior to RT (2040)	
ED visit or hospitalization	1174 (57.5%)
No ED visit or hospitalization	866 (42.5%)
Alberta Health Services Zone (n = 2038)	
South	114 (5.6%)
Calgary	1044 (51.2%)
Central	163 (8.0%)
Edmonton	527 (25.9%)
North	190 (9.3%)
Distance from Cancer Center (n = 2034)	
Less than 50 km	1715 (84.3%)
Greater than or equal to 50 km	319 (15.7%)
**(B)**	
**Subset Demographics**	**N(%)**
Rurality (n = 367)	
Urban	304 (82.8%)
Rural	63 (17.2%)
Karnofsky Performance Scale (n = 351)	
Greater than or equal to 70	159 (45.3%)
Less than 70	192 (54.2%)
Presence of Hepatic Metastases (n = 360)	
No	305 (84.7%)
Yes	55 (15.3%)
Presence of Brain Metastases (n = 361)	
No	313 (86.7%)
Yes	48 (13.3%)
Concurrent Systemic Therapy (n = 357)	
No	207 (58.0%)
Yes	150 (42.0%)
Inpatient Status at the Time of RT (n = 359)	
No	287 (79.9%)
Yes	72 (20.1%)
Coming from (n = 298)	
Home	231 (77.5%)
Hospital	58 (19.5%)
Long-Term Care	9 (3.0%)

^☨^ The greatest number of fractions prescribed to a patient at one treatment site.

**Table 2:** Univariate and multivariable logistic regression analysis of predictors of palliative intent radiation completion among 2040 patients treated with pRT.

		Univariate Analysis			Multivariate Analysis		
Variable		OR	95% CI	*p*-Value	OR	95% CI	*p*-Value
Age (years)		1.00	0.99–1.01	0.94	1.00	0.99–1.01	0.80
Sex	Male (n = 1085)	Ref			Ref		
	Female (n = 955)	0.86	0.64–1.15	0.31	0.786	0.577–1.07	0.13
Multiple Malignancies	No (n = 1720)	Ref			Ref		
	Yes (n = 320)	0.66	0.46–0.94	0.02	0.64	0.43–0.94	0.02
Retreatment	No (n = 1800)	Ref			Ref		
	Yes (n = 240)	1.46	0.87–2.44	0.15	1.48	0.87–2.53	0.15
Life Expectancy Estimate	<1 month (n = 14)	Ref			Ref		
	1–3 months (n = 210)	2.17	0.44–10.6	0.34	2.88	0.53–15.6	0.22
	3–6 months (n = 439)	1.40	0.30–6.40	0.67	1.89	0.37–9.72	0.45
	6–12 months (n = 732)	1.32	0.29–6.01	0.72	1.55	0.30–7.93	0.60
	>12 months (n = 645)	1.94	0.42–8.91	0.40	1.84	0.35–9.58	0.47
Proximity to Death	<30 days (n = 157)	Ref			Ref		
	30–59 days (n = 234)	1.81	1.07–3.08	0.03	2.20	1.26–3.85	0.01
	60–89 days (n = 218)	2.03	1.17–3.52	0.01	2.61	1.46–4.69	0.001
	90+ days (n = 1431)	3.70	2.42–5.66	<0.0001	5.02	3.08–8.20	<0.0001
Distance to RT Site	<50 KM (n = 1715)	Ref			Ref		
	>= 50 KM (n = 319)	0.92	0.62–1.36	0.67			
Highest RT Fractions Planned to Any Site	1 Fraction (n = 388)	Ref			Ref		
	2–5 Fractions (n = 1181)	0.18	0.08–0.39	<0.0001	0.17	0.08–0.38	<0.0001
	6–10 Fractions (n = 391)	0.09	0.04–0.19	<0.0001	0.08	0.03–0.17	<0.0001
	≥11 Fractions (n = 80)	0.12	0.04–0.31	<0.0001	0.09	0.03–0.26	<0.0001
ED Visit/Hospital Admission	ED Visit/Hospital Admit (n = 1174)	Ref			Ref		
	no ED visit or hospital Admit (n = 866)	1.56	1.14–2.13	0.005	1.24	0.89–1.74	0.21
SBRT/SRS	No (n = 1916)	Ref			Ref		
	Yes (n = 124)	4.54	1.43–14.4	0.01	3.75	1.14–12.3	0.03

**Table 3:** Univariate and multivariable logistic regression analysis of predictors of RT completion in Alberta among a subset of 367 patients prescribed pRT.

		Univariate Analysis			Multivariate Analysis		
Variable		OR	95% CI	*p*-Value	OR	95% CI	*p*-Value
Age (years)		1.01	0.98–1.04	0.47	1.01	0.98–1.04	0.55
Sex	Male (n = 163)	Ref.			Ref.		
	Female (n = 204)	0.67	0.34–1.34	0.26	0.64	0.27–1.34	0.28
Dyspnea	No (n = 140)	Ref.					
	Yes (n = 156)	1.03	0.48–2.19	0.94			
Anorexia	No (n = 132)	Ref.					
	Yes (n = 161)	1.10	0.50–2.42	0.81			
PaP Score	Score 0 to 5.5 (n = 336)	Ref.					
	>=Score 5.6 (n = 31)	1.27	0.42–3.85	0.67			
Hepatic Metastases	No (n = 305)	Ref.			Ref.		
	Yes (n = 55)	2.57	1.19–5.56	0.02	2.59	1.05–6.37	0.04
Brain Metastases	No (n = 313)	Ref.					
	Yes (n = 48)	1.50	0.62–3.62	0.37			
Systemic Therapy	No (n = 207)	Ref.			Ref.		
	Yes (n = 150)	0.66	0.33–1.33	0.25	0.89	0.39–2.02	0.77
Coming From	Home (n = 231)	Ref.					
	Hospital (n = 58)	1.13	0.14–9.45	0.91			
	Long-Term Care (n = 9)	0.78	0.09–7.11	0.83			
Multiple Malignancies	No (n = 244)	Ref.			Ref.		
	Yes (n = 123)	0.70	0.35–1.37	0.30	0.56	0.26–1.18	0.13
Retreatment	No Retreatment (n = 321)	Ref.			Ref.		
	Yes Retreatment (n = 46)	1.81	0.53–6.14	0.34	2.63	0.68–10.25	0.16
Proximity to Death	<30 days (n = 21)	Ref.			Ref.		
	30–59 days (n = 41)	3.70	0.91–15.01	0.07	6.61	1.23–35.61	0.03
	60–89 days (n = 39)	1.018	0.31–3.30	0.98	1.40	0.32–6.01	0.66
	90+ days (n = 266)	5.51	1.91–15.92	0.002	8.18	2.08–32.13	0.003
Highest RT Fractions Planned to Any Site	1 (n = 80)	Ref.			Ref.		
	2–5 (n = 207)	0.41	0.12–1.43	0.16	0.20	0.04–0.95	0.04
	6–10 (n = 69)	0.13	0.04–0.47	0.002	0.05	0.01–0.25	<0.0001
	≥11 (n = 11)	0.18	0.03–1.19	0.08	0.06	0.01–0.55	0.013
ED Visit or Hospital Admission	ED Visit/Hospital Admission (n = 189)	Ref.					
	No ED/Hospital Admission (n = 178)	1.40	0.72–2.75	0.33			
SBRT	No SBRT (n = 347)	Ref.					
	SBRT (n = 20)	1.07	0.24–4.82	0.93	1.13	0.16–7.88	0.90

## Data Availability

Data will not be shared, as the ethics approval for this project does not permit for sharing of this data due to patient safety and confidentiality.

## Supplementary Materials

The following supporting information can be downloaded at https://www.mdpi.com/article/10.3390/curroncol30120730/s1, Figure S1: Futile radiotherapy at the end of life questionnaire embedded into the ARIA-RO physician prescribing workflow.

## Appendix A

The data captured in the text were analyzed in two ways.

Aggregate questionnaire data, which include all RT prescriptions associated with a completed questionnaire between November 2017 and January 2020, are used in Figure 1 and Figure 2. In this dataset, patients could have multiple questionnaires completed on the same day for separate radiation prescriptions to separate sites (e.g., lung and brain). Subsequent visits of patients were also represented in this dataset.

Aggregate data were also sorted into patient-specific data, where the first recorded visit of each unique patient was determined. For each sorted patient visit, additional data were captured using the methods described above.
