# Supplementary material for: An Analysis of Clinical and Systemic Factors Associated with Palliative Radiotherapy Delivery and Completion at the End of Life in Alberta, Canada
Source: Curr Oncol. 2023 Nov 21;30(12):10043–56. doi: 10.3390/curroncol30120730 (PMC10742975; doi:10.3390/curroncol30120730)
Supplement: Supplementary file 1 [file curroncol-30-00730-s001.zip › curroncol-2694022-supplementary.pdf]

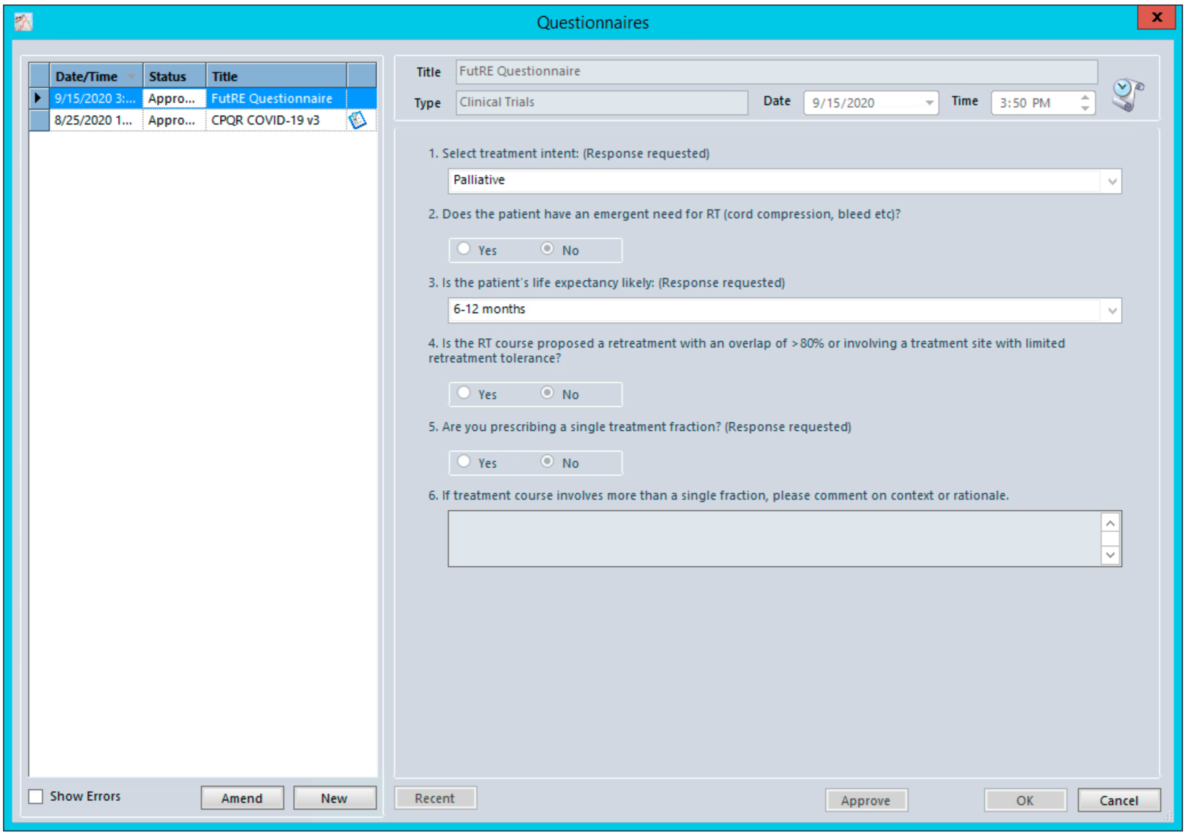

Figure S1. Futile radiotherapy at the end of life questionnaire embedded into the ARIA-RO physician prescribing workflow.
